# Intestinal IgA Regulates Expression of a Fructan Polysaccharide Utilization Locus in Colonizing Gut Commensal Bacteroides thetaiotaomicron

**DOI:** 10.1128/mBio.02324-19

**Published:** 2019-11-05

**Authors:** Payal Joglekar, Hua Ding, Pablo Canales-Herrerias, Pankaj Jay Pasricha, Justin L. Sonnenburg, Daniel A. Peterson

**Affiliations:** a Department of Microbiology and Immunology, Stanford University School of Medicine, Stanford, California, USA; b Department of Pathology, Johns Hopkins University School of Medicine, Baltimore, Maryland, USA; c Center for Neurogastroenterology, Department of Medicine, Johns Hopkins University School of Medicine, Baltimore, Maryland, USA; Maurice Müller Laboratories, Department of Clinical Research (DKF), UVCM, University Hospital; University of Hawaii at Manoa

**Keywords:** *Bacteroides thetaiotaomicron*, immunoglobulin A, diet, fructan, microbiota

## Abstract

Gut-derived immunoglobulin A (IgA) is the most abundant antibody secreted in the gut that shapes gut microbiota composition and functionality. However, most of the microbial antigens targeted by gut IgA remain unknown, and the functional effects of IgA targeting these antigens are currently understudied. This study provides a framework for identifying and characterizing gut microbiota antigens targeted by gut IgA. We developed a small intestinal *ex vivo* culture assay to harvest lamina propria IgA from gnotobiotic mice, with the aim of identifying antigenic targets in a model human gut commensal, Bacteroides thetaiotaomicron VPI-5482. Colonization by B. thetaiotaomicron induced a microbe-specific IgA response that was reactive against diverse antigens, including capsular polysaccharides, lipopolysaccharides, and proteins. IgA against microbial protein antigens targeted membrane and secreted proteins with diverse functionalities, including an IgA specific against proteins of the polysaccharide utilization locus (PUL) that are necessary for utilization of fructan, which is an important dietary polysaccharide. Further analyses demonstrated that the presence of dietary fructan increased the production of fructan PUL-specific IgA, which then downregulated the expression of fructan PUL in B. thetaiotaomicron, both *in vivo* and *in vitro*. Since the expression of fructan PUL has been associated with the ability of B. thetaiotaomicron to colonize the gut in the presence of dietary fructans, our work suggests a novel role for gut IgA in regulating microbial colonization by modulating their metabolism.

## Abstract (precis)

Given the significant impact that gut microbes have on our health, it is essential to identify key host and environmental factors that shape this diverse community. While many studies have highlighted the impact of diet on gut microbiota, little is known about how the host regulates this critical diet-microbiota interaction. In our present study, we discovered that gut IgA targeted a protein complex involved in the utilization of an important dietary polysaccharide: fructan. While the presence of dietary fructans was previously thought to allow unrestricted growth of fructan-utilizing bacteria, our work shows that gut IgA, by targeting proteins responsible for fructan utilization, provides the host with tools that can restrict the microbial utilization of such polysaccharides, thereby controlling their growth.

## INTRODUCTION

IgA is the most abundant immunoglobulin isotype secreted in the gut by mammals in response to microbial colonization (1). It has long been recognized as a first line of defense against enteric pathogens and, by the process of immune exclusion, limits microbial epithelial penetration to prevent systemic infection (2). It is now appreciated that the IgA plays a much broader role in intestinal homeostasis that extends beyond classical pathogens (3) and into regulation of the commensal gut microbiota (4–6).

Using mouse models that either lack IgA (AID^–/–^) (5) or carry an altered IgA repertoire (*Pdcd1*^−/−^) (4), previous studies have demonstrated that the lack of a “normal” IgA resulted in microbial dysbiosis in the small intestine. Intriguingly, a study comparing the gut microbiota of *Rag1^−/−^* to that of *Rag1^+/+^* mice revealed a selective decrease in the relative abundance of *Lactobacillales* and *Enterobacterales* accompanied by an enrichment of *Verrucomicrobiales* in *Rag1^−/−^* mice (7). This indicates a high degree of specificity of intestinal adaptive immune response (presumably including IgA), which would allow the host to selectively target only certain microbial members. In accordance with this, studies have found that IgA coating of gut commensals is highly variable, with only a limited fraction displaying high levels of IgA binding (8–10). Interestingly, IgA differentially targeted even closely related bacterial strains (Bacteroides fragilis) (8), demonstrating the discriminatory potential of this IgA response. IgA binding was not limited to species that were potentially colitogenic (8) but extended to those that were isolated from healthy individuals and were not associated with any enteropathy (Akkermansia muciniphila and Clostridium scindens) (9). Further extending the relevance of IgA to humans, a recent study comparing the fecal microbiota of patients with IgA deficiency to that of healthy controls showed a moderately dysbiotic gut microbiota with disrupted microbial networks in the patient cohort (11).

All these studies demonstrate the complexity of the IgA response and highlight the need to identify antigenic targets and underlying microbial pathways in order to understand how IgA selectively regulates the physiology of certain members of our microbiota. Previous attempts to find natural IgA targets have identified structural and functional components of commensal bacteria, including lipopolysaccharide (LPS)-O antigens, capsular antigens, and flagellar antigens that bind gut IgA (12–14). For some of these epitopes that were shown to be determinants of bacterial fitness *in vivo*, the presence of specific IgAs resulted in the selection of a bacterial population with lower expression of epitope-encoding genes (12, 13). Another study reported the role of IgA in promoting gut microbiota symbiosis by specific upregulation of a polysaccharide utilization locus in colonizing Bacteroides thetaiotaomicron via nonspecific IgA interaction (15). However, barring these examples of low-affinity or nonspecific interactions, currently, little is known about the microbial antigens that prime a specific IgA response.

To address this paucity of knowledge, we used a gnotobiotic mouse model monocolonized with a prominent human gut commensal, Bacteroides thetaiotaomicron VPI-5482. We developed an *ex vivo* small intestinal culture supernatant (SI culture supernatant) assay to harvest murine gut IgA, which enabled monitoring of the small intestinal IgA response against colonizing B. thetaiotaomicron. SI culture supernatants were used for the screening of a B. thetaiotaomicron genomic expression library to identify bacterial protein antigens. Of the multiple putative IgA targets found in our screen, proteins involved in the utilization of dietary polysaccharides (pectin and fructans) were identified as novel targets. By focusing on the well-characterized fructan utilization proteins (16), we demonstrate that the specific IgA response against these proteins was generated only in the presence of dietary fructans, which are known inducers of the fructan utilization locus in B. thetaiotaomicron. Generation of this response corresponded with a lowered expression of the locus in colonizing B. thetaiotaomicron. Considering the significant role of fructans and, in general, of dietary polysaccharides in shaping gut microbiota dynamics, our present work provides an important function for gut IgA in regulating the metabolism of gut microbes.

## RESULTS

### B. thetaiotaomicron induced a specific gut IgA response upon colonization of germfree mice.

**FIG 1:**
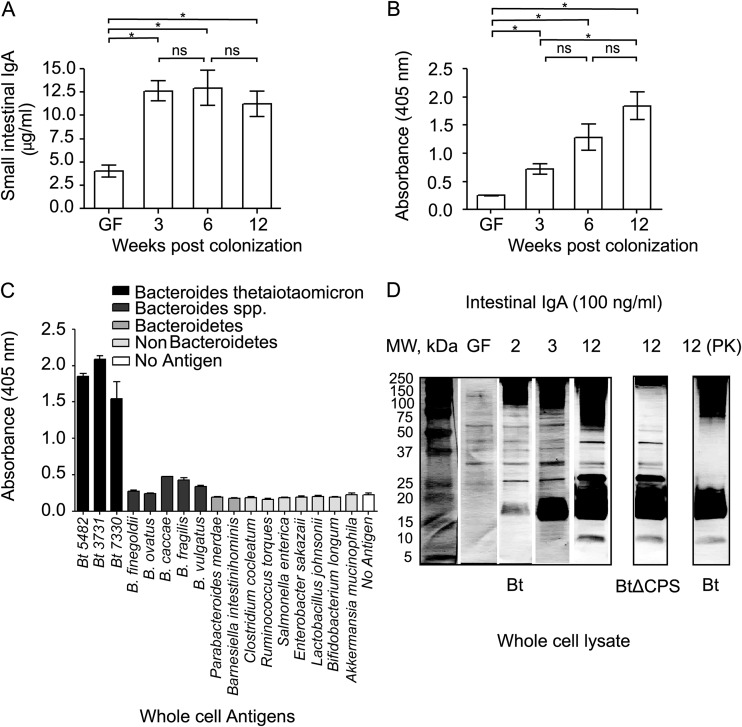
Gut colonization of germfree C57BL/6J mice with B. thetaiotaomicron elicits a bacterium-specific gut IgA response. (A) Isotype ELISA for measuring total IgA in SI culture supernatants. GF, germfree; 3, 6, and 12 represent weeks postmonocolonization with B. thetaiotaomicron. (B) Specific ELISA for measuring IgA reactive to B. thetaiotaomicron whole-cell antigens. *, *P* < 0.05. (C) Specific ELISA to measure cross-reactivity of B. thetaiotaomicron-induced gut IgA response against other gut microbes, including closely related B. thetaiotaomicron strains (labeled as Bt), bacteria from the same genus, the same phylum, and different phyla represented by shades of gray. (D) Western blot analyses of SI culture supernatants using B. thetaiotaomicron (Bt) and B. thetaiotaomicron ΔCPS (BtΔCPS) whole-cell lysate to detect B. thetaiotaomicron antigens that bind gut IgA. Number above each blot represents the week at which supernatant was harvested after B. thetaiotaomicron monocolonization. PK, proteinase K treatment; MW, molecular weight.

B. thetaiotaomicron, a prominent human gut bacterium with a fully sequenced genome, induces a homeostatic, noninflammatory intestinal IgA response upon colonization of germfree mice and serves as a good candidate to study the gut immune response to colonizing commensals (13–15, 17). To identify B. thetaiotaomicron antigens that prime this response, we orally gavaged B. thetaiotaomicron into 6- to 12-week-old germfree C57BL/6J mice that were fed a standard diet (STD diet) rich in microbiota-accessible carbohydrates (MACs) (18). The small intestinal lamina propria has the largest population of IgA^+^ plasma cells, which results in high levels of free and microbiota-bound IgAs within this gut compartment (19). We therefore developed an *ex vivo*, small intestinal lamina propria fragment culture system in order to harvest supernatants containing IgA secreted by the gut-resident plasma cells. Similar attempts were previously reported for obtaining IgA from murine Peyer’s patches and human intestinal biopsy samples (20–22). Small intestinal (SI) culture supernatants were generated from germfree controls and B. thetaiotaomicron monocolonized mice at multiple weeks postcolonization, and the amount of IgA produced from an individual small intestine was quantified using isotype enzyme-linked immunosorbent assay (ELISA; *n* = 3 to 4 biological replicates per condition). In line with previous studies, isotype ELISA showed a significant increase in total IgA upon B. thetaiotaomicron colonization (mean ± standard error of the mean [SEM] total IgA expressed in μg/ml: germfree, 4.023 ± 0.660; B. thetaiotaomicron monocolonized for 3 weeks, 12.61 ± 1.068; *P* = 0.002 by Student’s *t* test) (Fig. 1A) (23). ELISA using whole-cell antigens revealed an increase in the anti-B. thetaiotaomicron IgA response at week 3 (mean ± SEM B. thetaiotaomicron-specific IgA expressed as optical density [OD; 405 nm]: germfree, 0.250 ± 0.008; B. thetaiotaomicron monocolonized for 3 weeks, 0.718 ± 0.092; *P* = 0.007 by Student’s *t* test) (Fig. 1B). However, unlike total IgA, whose levels plateaued at week 3 (mean ± SEM total IgA expressed in μg/ml: 6 weeks, 12.93 ± 1.894; 12 weeks, 11.21 ± 1.388), the anti-B. thetaiotaomicron IgA response continued to increase past week 3 through to week 12 (mean ± SEM B. thetaiotaomicron-specific IgA expressed as OD [405 nm]: 6 weeks, 1.283 ± 0.232; 12 weeks, 1.843 ± 0.250). Previous studies have shown that even transient gut colonization of germfree mice causes a rapid increase in the number of IgA-secreting plasma cells in the lamina propria, which leads to an overall increase in total IgA in ex-germfree mice (23). Furthermore, colonizing microbiota trigger somatic hypermutation in these plasma cells, resulting in antigenic selection of the IgA repertoire overtime, which is reflected in the progressive increase in the B. thetaiotaomicron-specific IgA response in Fig. 1B (24). To test the ability of this specific IgA response to discriminate between B. thetaiotaomicron and other microbes, we carried out an ELISA using whole-cell antigens derived from a diverse array of gut microbes grown *in vitro*. This showed that the polyclonal IgA using week-12 SI culture supernatants from B. thetaiotaomicron-monocolonized mice was highly specific to Bacteroides thetaiotaomicron strains, with low cross-reactivity even to closely related *Bacteroides* (Fig. 1C).

Isotype and antigen-specific ELISAs were conducted on SI culture supernatants and sera obtained at week 12 to confirm that the B. thetaiotaomicron-specific antibody response was local and limited to the gut lamina propria and was predominantly driven by IgA (see Fig. S1A to D in the supplemental material). In addition to SI culture supernatant carrying B. thetaiotaomicron-specific IgA, we observed serum IgM reactivity to B. thetaiotaomicron, consistent with previous reports showing the presence of antigen-reactive natural IgM antibodies that are generated in the sera of animals by endogenous ligands (2).

10.1128/mBio.02324-19.2 FIG S1 B. thetaiotaomicron-specific antibody response is predominantly driven by gut lamina propria restricted IgA. Isotype ELISA to measure total antibody isotypes in serum (A) and SI culture supernatants (B) at week 12 after B. thetaiotaomicron colonization. Specific ELISA using the above samples to measure B. thetaiotaomicron-specific IgA, IgM, and IgG isotypes in serum (C) and SI culture supernatants (D). Download FIG S1, PDF file, 0.1 MB. Copyright © 2019 Joglekar et al. 2019 Joglekar et al. This content is distributed under the terms of the Creative Commons Attribution 4.0 International license.

To visualize the type and size distribution of antigens, we carried out a Western blot analysis using B. thetaiotaomicron whole-cell lysate (Fig. 1D). SI culture supernatants were normalized to 100 ng/ml of total IgA and used as a source of primary antibody. Germfree SI culture supernatants displayed weak reactivity to B. thetaiotaomicron antigens, owing to natural IgAs present in the gut devoid of antigenic stimulation (10). There was a progressive change in the number and intensity of antigenic bands from germfree to week 12 postcolonization with B. thetaiotaomicron. The majority of antigenic bands were centered on 100 to 250 kDa and 17 to 20 kDa, in addition to two bands close to ∼25 kDa and ∼37 kDa. To characterize the type of antigens that bind gut IgA, we first carried out a Western blot analysis using a B. thetaiotaomicron mutant strain lacking all eight annotated capsular polysaccharide synthesis (CPS) loci (B. thetaiotaomicron ΔCPS) (25). This revealed that the high-molecular-weight fraction corresponded to B. thetaiotaomicron capsular antigens. To characterize the nature of the remainder antigens, we carried out proteinase K treatment of the blotting membrane before probing with week-12 SI culture supernatants. Antigenic bands running between ∼20 to 100 kDa were degraded by proteinase K, confirming their proteinaceous nature. A large proportion of low-molecular-weight bands were resistant to proteinase K and possibly represent lipopolysaccharide (LPS) antigens, which are known targets of IgA (14, 26).

Our data show that B. thetaiotaomicron colonization induced a gut mucosa-restricted polyclonal yet specific IgA response against a variety of B. thetaiotaomicron antigens, which could distinguish the colonizing bacterium and its closely related strains from other gut commensals.

### Gut mucosal IgA targeted dietary nutrient utilization loci in B. thetaiotaomicron.

**FIG 2:**
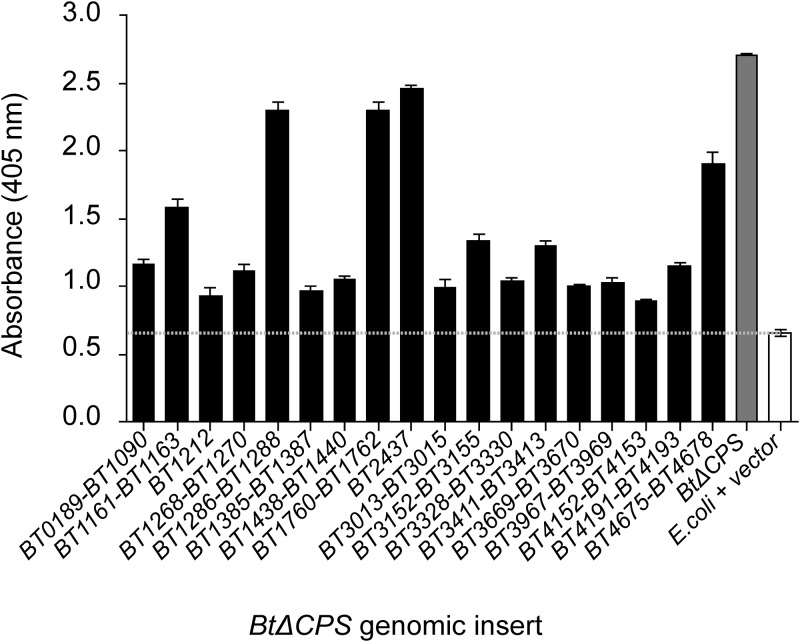
Identification of B. thetaiotaomicron antigens that prime a specific gut IgA response. Antigen-specific high-sensitivity ELISA using week-12 SI supernatants to confirm E. coli transformants carrying B. thetaiotaomicron ΔCPS genomic library inserts that bind gut IgA. B. thetaiotaomicron ΔCPS (BtΔCPS), positive control; E. coli+vector, negative control.

Our group previously identified specific gut IgA responses against B. thetaiotaomicron’s capsular and LPS antigens and demonstrated their role in gut homeostasis (13, 14). Here, we focused on B. thetaiotaomicron proteins that serve as natural targets of gut IgA. The rationale for doing so was to find functional rather than structural antigens of IgA, thus allowing identification of immune targets that govern B. thetaiotaomicron metabolism *in vivo*. With this purpose in mind, we generated a B. thetaiotaomicron ΔCPS random genomic expression library (lacking genes encoding all eight B. thetaiotaomicron capsules) in a laboratory strain of Escherichia coli, using a previously described protocol (27). Since week-12 SI culture supernatants had the highest concentration of polyclonal IgA, these were pooled and used as an IgA source for screening the expression library by a standard colony dot blot assay. Around 10^4^ colonies were screened, and B. thetaiotaomicron ΔCPS genomic inserts from the IgA reactive library clones were sequenced using plasmid specific primers (see Table S1). A total of 18 unique clones were confirmed to be positive using a biotin-tyramide amplification-based high-sensitivity ELISA (Fig. 2, Table S2; see also Text S1 and Fig. S3 for details). A total of 49 genes were represented in these 18 clones, the majority of which (41 genes) encoded proteins predicted to be noncytoplasmic in localization. Around 30% of the genes were hypothetical with unknown function. Most of the other genes with a predicted function were putative homologs within uncharacterized loci either encoding efflux pump proteins or involved in the utilization of polyamines, proteins, and glycans as nutrients by B. thetaiotaomicron. Interestingly, among the nutrient pathways targeted by gut IgA, two loci are involved in the utilization of dietary microbiota-accessible carbohydrates (MACs) (fructan, *BT1760-BT1762*, and pectin, *BT4152-BT4153*). Dietary MACs are one of the most important drivers of gut microbiota composition and biogeography (28). Furthermore, the metabolism of dietary MACs by gut microbes is an important source of host-utilizable metabolites such as short-chain fatty acids, which have a multitude of effects on host physiology (29). Given their significant effect on gut microbiota homeostasis, it is logical to hypothesize the host regulates dietary MAC utilization by gut commensals in order to control gut microbiota composition. In line with this idea, a recent finding reported a diet-dependent antigen from B. thetaiotaomicron (BT4295) to be a specific target of host CD4^+^ T cell response in the murine gut (17). To our knowledge, these dietary MAC utilization loci have not been implicated in bacterial virulence and thus could represent true commensal targets of gut IgA surveillance.

10.1128/mBio.02324-19.1 TEXT S1 Detailed materials and methods and development of high-sensitivity ELISA. Download Text S1, PDF file, 0.1 MB. Copyright © 2019 Joglekar et al. 2019 Joglekar et al. This content is distributed under the terms of the Creative Commons Attribution 4.0 International license.

10.1128/mBio.02324-19.4 FIG S3 Development of a high-sensitivity ELISA. (A) A cartoon depicting only the additional steps performed in the high-sensitivity ELISA. HRP, 2° antibody conjugated horseradish peroxidase; BioTy, biotinylated-tyramine; A, avidin. See text for details. (B) A comparison of ELISAs using serial dilutions of 2° anti-IgA-HRP with various dilutions of biotinylated-tyramine. Each well was coated with a fixed concentration of purified mouse IgA. (C) Serial dilutions of 1° purified mouse IgA tested with various dilutions of biotinylated-tyramine. Plates were coated with a fixed concentration of goat anti-mouse Ig. (D) Serial dilutions of culture supernatants from hybridoma cells secreting 1° B. thetaiotaomicron specific monoclonal IgA (260.8), tested in presence of B. thetaiotaomicron (specific antigen) and Bacteroides sartorii (*Bs*, nonspecific antigen). The reactions were carried out with and without BioTy amplification. (E) ELISA using fecal supernatants as a source of 1° antibody generated from gnotobiotic mice that were either germfree or monocolonized with B. thetaiotaomicron. GαM, goat anti-mouse Ig. Download FIG S3, PDF file, 0.4 MB. Copyright © 2019 Joglekar et al. 2019 Joglekar et al. This content is distributed under the terms of the Creative Commons Attribution 4.0 International license.

10.1128/mBio.02324-19.5 TABLE S1 List of primers used in this study. Download Table S1, PDF file, 0.1 MB. Copyright © 2019 Joglekar et al. 2019 Joglekar et al. This content is distributed under the terms of the Creative Commons Attribution 4.0 International license.

10.1128/mBio.02324-19.6 TABLE S2 Genes contained within the B. thetaiotaomicron ΔCPS genomic library inserts carrying putative epitopes recognized by polyreactive gut IgA. Download Table S2, PDF file, 0.1 MB. Copyright © 2019 Joglekar et al. 2019 Joglekar et al. This content is distributed under the terms of the Creative Commons Attribution 4.0 International license.

Based on previous knowledge and our current IgA screen data, we set out to test whether expression of these dietary MAC utilization loci were being regulated by the host via gut IgA. Of the two loci detected in our preliminary assay, we focused on the fructan utilization pathway primarily for two reasons. (i) The fructan locus induced a significantly higher IgA response than the pectin locus as confirmed by ELISA (Fig. 2), and (2) fructan utilization by B. thetaiotaomicron is carried out by a single well-characterized locus (16) compared to the complex regulation of multiple loci needed for pectin degradation (30). We presumed that these criteria would make it easier to monitor the expression of the fructan locus in the presence of specific IgA.

### Intestinal IgA regulates the expression of the fructan operon in B. thetaiotaomicron.

**FIG 3:**
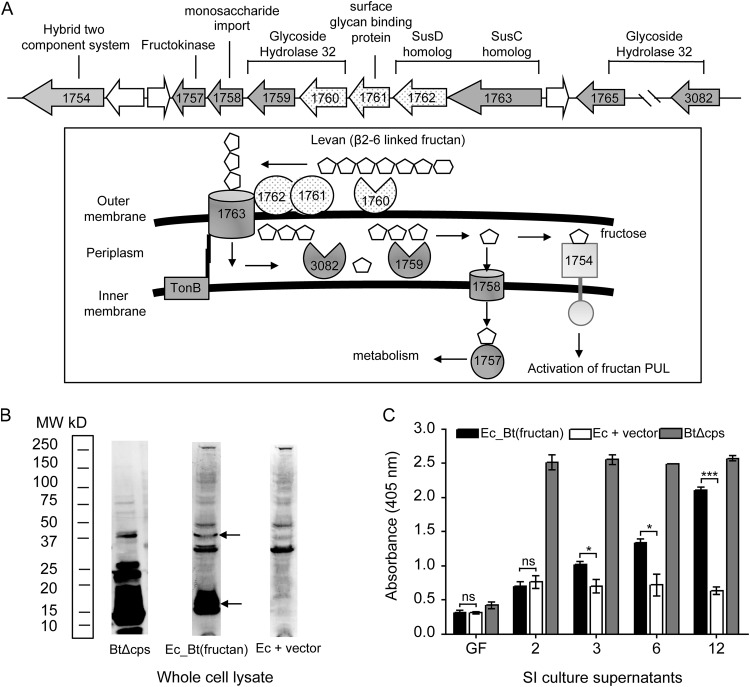
Proteins encoded by B. thetaiotaomicron’s fructan PUL are targets of gut IgA when mice are fed the STD diet. (A, top) Fructan PUL as encoded by the B. thetaiotaomicron VPI-5482 genome. (Bottom) Predicted model for levan (β2-6-linked fructan) utilization by B. thetaiotaomicron. The PUL is upregulated in the presence of monomeric fructose. Gene and protein symbols filled with dots were part of the B. thetaiotaomicron ΔCPS genomic insert that binds gut IgA. (B) Western blot analysis using week-12 SI culture supernatant and whole-cell lysate of library clone Ec_B. thetaiotaomicron(fructan) to confirm gut IgA reactivity. BtΔCPS, positive control; Ec_Bt(fructan), E. coli transformant carrying a partial B. thetaiotaomicron fructan PUL (*BT1760*, *BT1761*, *BT1762*); Ec+vector, negative control; MW, molecular weight. (C) High-sensitivity ELISA to measure kinetics of anti-B. thetaiotaomicron(fructan) IgA generation upon B. thetaiotaomicron monocolonization.

Genes *BT1760*, *BT1761*, and *BT1762* encoded by the B. thetaiotaomicron genome are part of a polysaccharide utilization locus (PUL) involved in the degradation and uptake of β2-6-linked fructans, such as levan (Fig. 3A). These genes encode a glycoside hydrolase family 32 enzyme (BT1760), a lipoprotein (BT1761), and a SusD homolog (BT1762), which, along with a SusC homolog (BT1763), form a cell surface complex for the hydrolysis and outer membrane transport of levan. Monomeric fructose acts as an inducer for the fructan PUL (16). To confirm the presence of specific IgA targeting this transport machinery [referred to as anti-B. thetaiotaomicron(fructan) IgA], we performed a Western blot analysis using whole-cell lysate of the E. coli transformant carrying the partial B. thetaiotaomicron fructan locus: E. coli pZE21-MCS-1-*BT1760-BT1762* [referred to as Ec_B. thetaiotaomicron(fructan)] using pooled week-12 SI culture supernatants. Western blotting confirmed the presence of IgA-reactive antigens that were absent in the background E. coli strain (Fig. 3B). We next monitored the kinetics of the anti-B. thetaiotaomicron(fructan) IgA response using a high-sensitivity ELISA as described before (Fig. 3C). Germfree supernatants did not react against Ec_B. thetaiotaomicron(fructan), suggesting that anti-B. thetaiotaomicron(fructan) IgA is not present prior to B. thetaiotaomicron colonization. We only detected anti-B. thetaiotaomicron(fructan) IgA in SI culture supernatants harvested at week 3 and beyond from B. thetaiotaomicron-monocolonized mice.

**FIG 4:**
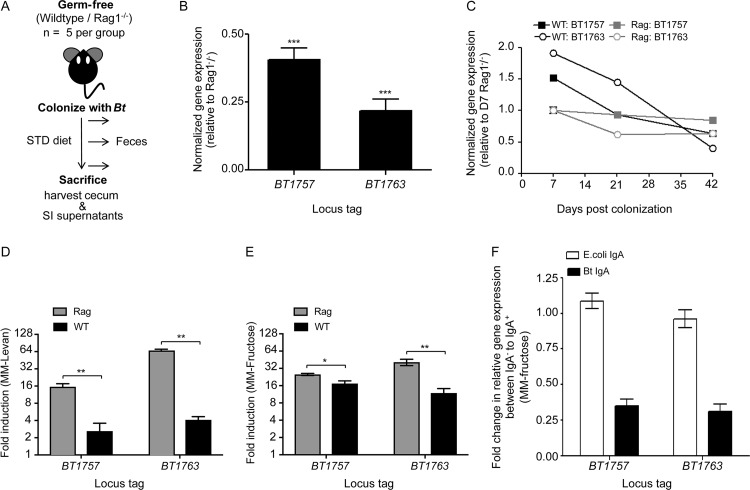
B. thetaiotaomicron*’s* fructan PUL is downregulated in WT mice relative to that in *Rag1^−/−^* mice. (A) Experimental design for *in vivo* experiment. (B) Average expression of fructan PUL in the ceca of WT mice relative to that in *Rag1^−/−^* mice at week 6 postcolonization. ***, *P* < 0.0001. (C) Time course of fructan PUL gene expression in WT and *Rag1^−/−^* mice feces. Normalized data are shown relative to PUL expression at day 7 in *Rag1^−/−^* mice. qRT-PCR analyses to measure expression of the fructan PUL in MM-levan (D) and MM-fructose (E) in the presence of SI culture supernatant from B. thetaiotaomicron-monocolonized WT or *Rag1^−/−^* mice (week 6). Expression is relative to that in MM-glucose. *, *P* < 0.01; **, *P* < 0.001. (F) Fold change in the expression of the fructan PUL in MM-fructose (relative to that in MM-G) under IgA^−^ and IgA^+^ conditions. B. thetaiotaomicron-specific IgA (Bt IgA), test; human E. coli-specific IgA, nonspecific IgA control (*n* = 3 biological replicates for each experiment).

Previous studies have demonstrated that IgA downregulates the expression of bacterial genes encoding IgA-specific epitopes (12, 13). To test if anti-B. thetaiotaomicron(fructan) IgA downregulates B. thetaiotaomicron’s fructan PUL, we carried out an *in vivo* experiment using gnotobiotic C57BL/6J wild-type (WT) and adaptive immune-deficient (*Rag1^−/−^*) mice (*n* = 5 mice per group) (Fig. 4A). Germfree mice fed the STD diet containing fructans were monocolonized with B. thetaiotaomicron. Feces were collected at regular intervals to monitor B. thetaiotaomicron colonization density and to quantify gene expression of the fructan PUL. Mice were sacrificed at week 6 for cecal and small intestinal contents and to harvest SI culture supernatants. Bacterial densities, as measured by plating anaerobically on brain heart infusion with 10% Sheep Blood (BHI-Blood) agar, were comparable between WT and *Rag1^−/−^* mice along the entire gut, with small intestine showing ∼100-fold lower B. thetaiotaomicron density than the cecum/feces (see Fig. S2A to C). We then set out to determine whether the presence of a functional adaptive immune system in WT mice impacts fructan PUL expression in B. thetaiotaomicron by using quantitative real-time PCR (qRT-PCR) analysis. Specifically, the expression of genes *BT1757* and *BT1763* was measured using gene-specific primers (Table S1), as they represent the first and the last gene, respectively, of the coregulated fructan operon (16). We observed a significant reduction in expression of the PUL in ceca of WT mice relative to that in *Rag1^−/−^* mice (average relative gene expression ± SEM: *BT1757*, 0.40 ± 0.09, *P* < 0.0001; *BT1763*, 0.22 ± 0.10, *P* < 0.0001 by Student’s *t* test) (Fig. 4B). In the time course analysis of anti-B. thetaiotaomicron(fructan) IgA by using an ELISA, there was a lack of an anti-B. thetaiotaomicron(fructan) IgA at week 2 after B. thetaiotaomicron colonization. We reasoned that if anti-B. thetaiotaomicron(fructan) IgA is a significant factor contributing to PUL expression differences at week 6, then earlier time points should have similar expression between the WT and *Rag1^−/−^* mice, with downregulation occurring only at week 3 and beyond in WT mice. To test this, we carried out qRT-PCR analysis on fecal samples collected at day 7 (week 1) [anti-B. thetaiotaomicron(fructan) IgA negative] and day 21 (week 3) and day 42 (week 6) [both anti-B. thetaiotaomicron(fructan) IgA positive] postcolonization. Expression of *BT1757* and *BT1763* was calculated relative to a normalized expression value of “1” at day 7 in *Rag1^−/−^* feces (Fig. 4C). We observed a significant reduction in the relative gene expression levels in WT mice from week 1 to week 6 (average relative gene expression ± SEM: *BT1757*, 1.52 ± 0.09 to 0.64 ± 0.07, *P* < 0.0001; *BT1763*, 1.91 ± 0.11 to 0.40 ± 0.07, *P* < 0.0001). Relative to that in the WT, the reduction in the fructan PUL expression was much weaker in *Rag1^−/−^* mice (average relative gene expression ± SEM: *BT1757*, 1.00 ± 0.11 to 0.85 ± 0.08, *P* = 0.034; *BT1763*, 1.00 ± 0.12 to 0.63 ± 0.10, *P* = 0.0007 by Student’s *t* test). This suggested a putative role of anti-B. thetaiotaomicron(fructan) IgA in the downregulation of the PUL.

10.1128/mBio.02324-19.3 FIG S2 B. thetaiotaomicron colonizes to similar densities irrespective of the host genotype and diet as determined by plating dilutions of intestinal contents on BHI-blood agar. Bacterial densities in small intestines and ceca were monitored at week 6 after B. thetaiotaomicron monocolonization. Download FIG S2, PDF file, 0.1 MB. Copyright © 2019 Joglekar et al. 2019 Joglekar et al. This content is distributed under the terms of the Creative Commons Attribution 4.0 International license.

Our *in vivo* experimental data showed that the gut mucosal immune system contributes to the downregulation of fructan PUL in WT mice. *Rag1^−/−^* mice, however, lack both mature B and T lymphocytes (31). The *in vivo* data were therefore indicative, but not confirmatory, of the role of IgA in regulating the expression of the fructan locus. To tease out the role of IgA, we quantified *in vitro* fructan gene expression of B. thetaiotaomicron grown in the presence of the SI culture supernatants harvested at week 6. These supernatants that lack any cells are enriched in molecules secreted by the intestinal tissue during *ex vivo* incubation. Minimal medium (MM) containing glucose (MM-G), fructose (MM-F), or levan (β2-6-linked fructan) (MM-L) as the sole carbon source were supplemented with WT and *Rag1^−/−^* SI culture supernatants (30% [vol/vol]). B. thetaiotaomicron was grown to mid-log phase, and gene-specific qRT-PCR analysis was carried out to measure fructan PUL expression in the presence of fructose or levan (induced state) relative to that with glucose (constitutive). Our data revealed reduced expression of the PUL in the presence of WT SI culture supernatant compared to that in *Rag1^−/−^* SI culture supernatant (*n* = 3) (average relative gene expression ± SEM with MM-F: *BT1757*-WT, 17.4 ± 1.9; *BT1757*-*Rag1^−/−^*, 25.0 ± 1.037, *P* < 0.004; *BT1763*-WT, 11.9 ± 2.3; *BT1763*-*Rag1^−/−^*, 41.0 ± 5.1, *P* < 0.006 by Student’s *t* test; with MM-L: *BT1757*-WT, 2.6 ± 1.0; *BT1757*-*Rag1^−/−^*, 15.5 ± 2.0, *P* < 0.005; *BT1763*-WT, 4.1 ± 0.6; *BT1763*-*Rag1^−/−^*, 65.6 ± 5.1, *P* < 0.001 by Student’s *t* test) (Fig. 4D and E). This strongly indicated that gut IgA regulates fructan PUL expression. We then carried out total IgA depletion of WT SI culture supernatants before adding them to B. thetaiotaomicron cultures. SI culture supernatants were also generated from gnotobiotic WT mice monocolonized with a human E. coli isolate; these served as nonspecific IgA control and were subjected to the same steps of total IgA depletion. qRT-PCR analysis was carried out on B. thetaiotaomicron grown in MM-F (relative to MM-G) containing IgA^−^ or IgA^+^ supernatants obtained from either B. thetaiotaomicron- or E. coli-monocolonized WT mice. This revealed attenuation of the PUL only in the presence of IgA from B. thetaiotaomicron-monocolonized mice (fold change in average relative gene expression between IgA^−^ to IgA^+^ ± SEM: *BT1757*, 0.35 ± 0.09; *BT1763*, 0.31 ± 0.09), with no significant difference in PUL expression in the presence of IgA obtained from E. coli-colonized mice (BT1757, 1.09 ± 0.09; BT1763, 0.95 ± 0.11) (Fig. 4F). Our combined *in vivo and in vitro* data revealed that anti-B. thetaiotaomicron(fructan) IgA is capable of modulating fructan PUL expression in B. thetaiotaomicron.

### Generation of anti-B. thetaiotaomicron(fructan) IgA response against B. thetaiotaomicron’s fructan PUL correlates with the presence of dietary fructans.

**FIG 5:**
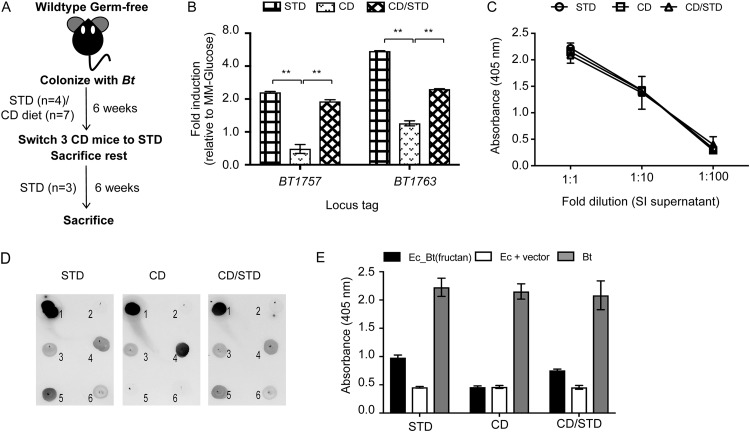
Generation of anti-B. thetaiotaomicron(fructan) IgA in B. thetaiotaomicron-colonized mice correlates with upregulation of the fructan locus in response to dietary fructan. (A) Experimental design for *in vivo* experiment. (B) Average relative expression of the fructan PUL in ceca of mice fed the STD and the CD at week 6 postcolonization and in ceca of mice that were switched from the CD (6 weeks) to the STD diet (6 weeks). Data are normalized to the expression of the PUL in MM-G. **, *P* < 0.001. (C) Specific ELISA for measuring IgA reactive to B. thetaiotaomicron whole-cell antigens in SI culture supernatants from mice fed STD, CD, and CD/STD. (D) Dot blot analyses using SI culture supernatants. Antigens used: 1, B. thetaiotaomicron (positive control); 2, E. coli plus vector (negative control); 3, *BT1161*-*BT1163*; 4, *BT1286*-*BT1288*; 5, *BT1760*-*BT1762*; 6, *BT2437*. (E) High-sensitivity ELISA to measure anti-B. thetaiotaomicron(fructan) IgA in SI culture supernatants in mice fed different diets.

Prior work has established a marked substrate-dependent upregulation of B. thetaiotaomicron’s fructan utilization PUL, with induced expression observed only in the presence of fructose and its polymers (16). This led us to hypothesize that generation of an anti-B. thetaiotaomicron(fructan) IgA response in B. thetaiotaomicron-monocolonized mice is a function of host diet. To test this, germfree mice were either fed the STD diet as before (*n* = 4) or a custom diet (CD) (*n* = 7) lacking any fructans. Mice were monocolonized with B. thetaiotaomicron and maintained on respective diets for 6 weeks postcolonization. At this point, all mice receiving the STD diet and 4 mice on the CD were sacrificed for further analysis. The remainder of the CD mice (*n* = 3) were then switched to the STD diet for an additional 6 weeks (Fig. 5A). Cecal B. thetaiotaomicron density was quantified as before and was independent of the host diet (Fig. S2D). Cecal fructan PUL expression was measured using qRT-PCR analysis and expressed relative to *in vitro* expression in MM-glucose (MM-G). Induced expression of the PUL was observed only in mice receiving the STD diet either from the start of the experiment or after a dietary switch from CD to STD, correlating with previous studies showing PUL activation with the STD diet (32) (average relative gene expression ± SEM for *BT1757*: STD, 2.3 ± 0.04; CD/STD, 1.9 ± 0.07, *P* < 0.01; for *BT1763*: STD, 5.5 ± 0.04; CD/STD, 2.4 ± 0.02, *P* < 0.01 by Student’s *t* test) (Fig. 5B). Host diet did not change the total gut IgA (data not shown) and did not affect the IgA response against B. thetaiotaomicron whole-cell antigens, as measured using a standard ELISA (Fig. 5C). To study if host diet affects the IgA response against specific B. thetaiotaomicron antigens, we carried out a dot blot assay using clones from the B. thetaiotaomicron ΔCPS genomic library that were targets of gut IgA (from Fig. 2). Of the 18 clones that reacted against week-12 SI supernatants, only 4 clones, *viz.*, *BT1161-BT1163*, *BT1286-BT1288*, *BT1760-BT1762* [Ec_B. thetaiotaomicron(fructan)], and *BT2437*, were reactive against week-6 SI supernatants harvested from STD diet-fed mice (Fig. 5D). A comparison with the CD showed that Ec_B. thetaiotaomicron(fructan) and the hypothetical protein *BT2437* did not react with SI supernatants harvested from these mice. Interestingly, a dietary switch to the STD diet restored reactivity against all 4 clones, showing that the gut IgA response against these B. thetaiotaomicron antigens was dependent on the host diet. IgA response against Ec_B. thetaiotaomicron(fructan) was then quantified using a high-sensitivity ELISA (Fig. 5E), which confirmed our dot blot results. Together, our dietary data showed that induced expression of the fructan PUL in B. thetaiotaomicron was associated with the generation of anti-B. thetaiotaomicron(fructan) IgA, specifically in mice that were fed dietary fructans.

## DISCUSSION

Gut IgA plays an important role in mucosal immunity and gut microbiota homeostasis (3); however, currently, there is a paucity of information about commensal antigens that are specific targets of this IgA. The tremendous diversity displayed by the human gut microbiota, which collectively harbors >10^5^ nonredundant genes (33), has made it experimentally challenging to identify important IgA-antigen pairs from a polyclonal gut IgA response. To circumvent this issue, we used a gnotobiotic system by colonizing the intestines of germfree C57BL/6J mice with only B. thetaiotaomicron. Our technique of using SI culture supernatants has some unique advantages over measuring IgA in either intestinal lavage or feces. In the latter case, the IgA profile may change due to host and bacterial proteolytic degradation, making accurate quantification of IgA produced difficult (34). Furthermore, a large amount of IgA is already bound to gut bacteria, making it difficult to identify the antigenic targets. *De novo* synthesis and secretion of IgA in a culture medium devoid of any antigenic stimulation eliminates these issues. This technique revealed that under the current environmental setup, IgA response was directed against extracellular and membrane proteins encoded by genes with diverse functionalities. Interestingly, many of these genes or their homologs are known to be upregulated by *Bacteroides in vivo*, indicating their role in colonization and survival in the gut, and thus warrant further investigation (32, 35–39). For our present work, we focused on a nutrient utilization pathway involved in fructan use, which to our knowledge, is a novel target for intestinal IgA. Fructans are fructose-based homopolymers abundant in plant-based diets (40). The inability of mammalian hosts to digest these polymers makes them a significant energy source for the gut bacteria. In fact, previous studies have established the significance of fructan use in B. thetaiotaomicron by showing that the fructan PUL confers a fitness advantage to B. thetaiotaomicron *in vivo* (38) and that fructose-containing carbohydrates are preferentially utilized by B. thetaiotaomicron over other carbon sources (32, 41). It is possible that by modulating the utilization of fructans by B. thetaiotaomicron, the host can potentially control B. thetaiotaomicron population in the gut and thereby maintain a stable gut community in the context of a complex microbiota. A recent report has suggested that humans have significant seasonal variations in diets, with specific food groups dominating our diet in certain seasons, and that these affect significant changes in proportional representations of the major bacterial groups in the gut microbiota (42). However, despite significant shifts in diets over time, the general composition of our gut microbiota is maintained. Our work proposes that gut IgA has a role in maintaining diversity of intestinal microbiota even in such seasonal variations of dietary restrictions and changes, and it does so by modulating the metabolism of specific microbes during seasonal dietary changes.

One caveat of our present work is the use of polyclonal IgA to study the effect of anti-B. thetaiotaomicron(fructan) IgA. In the absence of a monoclonal anti-B. thetaiotaomicron(fructan) IgA, it is impossible to conclusively state the role of this specific IgA in regulation of the fructan PUL in B. thetaiotaomicron. Another caveat is the use of standard and custom diets as complementary in the dietary study outlined in Fig. 5. These two diets differed beyond the presence of fructans, such as in the amount of vitamins and fats present. Also, the STD diet was complex and autoclavable, whereas the CD was refined and irradiated (see Materials and Methods and Table S3 in the supplemental material for diet composition).

10.1128/mBio.02324-19.7 TABLE S3 Composition of Teklad custom diet (TD.170584) used in the study. Download Table S3, PDF file, 0.1 MB. Copyright © 2019 Joglekar et al. 2019 Joglekar et al. This content is distributed under the terms of the Creative Commons Attribution 4.0 International license.

Despite these limitations, our present work identified a novel role for specific gut IgA through which the host can regulate nutrient utilization by colonizing gut microbes. Future studies using specific monoclonal IgAs should provide a thorough understanding of the underlying mechanism by which IgA targets these bacterial pathways, and its impact in shaping dietary preferences in colonizing gut commensals.

## MATERIALS AND METHODS

Brief methods are outlined. Please refer to Text S1 in the supplemental material for more details.

### Bacterial strains.

Most studies were carried out using the type strain Bacteroides thetaiotaomicron VPI-5482. A human gut E. coli isolated in our lab was used to generate nonspecific IgA-containing SI culture supernatant.

### *In vivo* experiments.

Six- to twelve-week-old germfree C57BL/6J mice were gavaged with ∼10^8^ CFU of B. thetaiotaomicron grown overnight in tryptone-yeast extract-glucose (TYG) (32). Diets used were either autoclaved microbiota-accessible carbohydrates (STD diet) (Labdiet JL Rat and Mouse/Auto 6F 5K67) or irradiated Teklad custom diet (TD.170584) (Table S3). All experiments were performed using protocols approved by the Johns Hopkins Animal Studies Committee (IACUC).

### Generation of small intestinal fragment lamina propria culture supernatant.

Briefly, small intestines obtained aseptically were cleaned, opened longitudinally, and minced into small pieces. Pieces were subjected to Dulbecco’s modified Eagle medium (DMEM) washes, incubation at 37°C in a citrate buffer solution, and final washes with DMEM plus 5% fetal bovine serum (FBS). Pieces were placed in DMEM plus 10% FBS for 36 h at 37°C, 5% CO_2_. Intestinal culture supernatants were harvested by centrifugation and maintained at 4°C.

### ELISA.

All ELISAs were performed using previously published protocols (13) in 96-well plates. The high-sensitivity ELISA involved using a biotin-tyramide amplification step at the detection stage. Refer to Text S1 for high-sensitivity ELISA details.

### Western blot analysis.

Western blot analysis was carried out using standard protocols. B. thetaiotaomicron grown overnight in TYG medium was used to obtain whole-cell lysate. Polyclonal IgA present in SI culture supernatants (100 ng/ml) was used as a source of primary antibody. For the proteinase K assay, after protein transfer onto the polyvinylidene difluoride (PVDF) membrane, the membrane was incubated with 5 μg/ml proteinase K for 30 min at 37°C (2).

### Construction of B. thetaiotaomicron ΔCPS genomic library.

Random genomic expression library of B. thetaiotaomicron ΔCPS was generated using E. coli MegaX DH10B T1R Electrocomp Cells (Invitrogen) as described before (27).

### Colony dot blot analysis.

Colony dot blot assay was performed using a previously published protocol (14).

### Quantitative real-time PCR.

RNA was extracted from B. thetaiotaomicron cultures, cecal contents, or feces stored in RNAprotect (Qiagen) using the RNeasy kit (Qiagen). cDNA was generated using SuperScript II reverse transcriptase (Invitrogen). qRT-PCR was performed using gene-specific primers (Table S1) with Brilliant III Ultra-Fast SYBR green QPCR reaction mixture (Agilent) in a Bio-Rad CFX96 instrument. 16S rRNA sequences were used for normalization, and fold changes were calculated using the threshold cycle (ΔΔ*C_T_*) method.

### *In vitro* growth using SI culture supernatant.

*Bacteroides* minimal medium supplemented with glucose, fructose, or levan (0.5% [wt/vol]) and containing SI culture supernatants (30% [vol/vol]) was inoculated (1:50) using a freshly grown culture of B. thetaiotaomicron in TYG. Cultures were incubated anaerobically at 37°C and harvested at mid-log phase for RNA isolation.

### IgA depletion.

SI culture supernatants harvested from C57BL/6J mice monocolonized with either B. thetaiotaomicron or E. coli were used for IgA depletion. Biotinylated goat anti-mouse IgA (Southern Biotech) was used to deplete IgA using an EasySep Mouse Streptavidin RapidSpheres isolation kit.
