# Supplementary figures and images for: Intestinal IgA Regulates Expression of a Fructan Polysaccharide Utilization Locus in Colonizing Gut Commensal Bacteroides thetaiotaomicron
Source: mBio. 2019 Nov 5;10(6):e02324-19. doi: 10.1128/mBio.02324-19 (PMC6831775; doi:10.1128/mBio.02324-19)

A

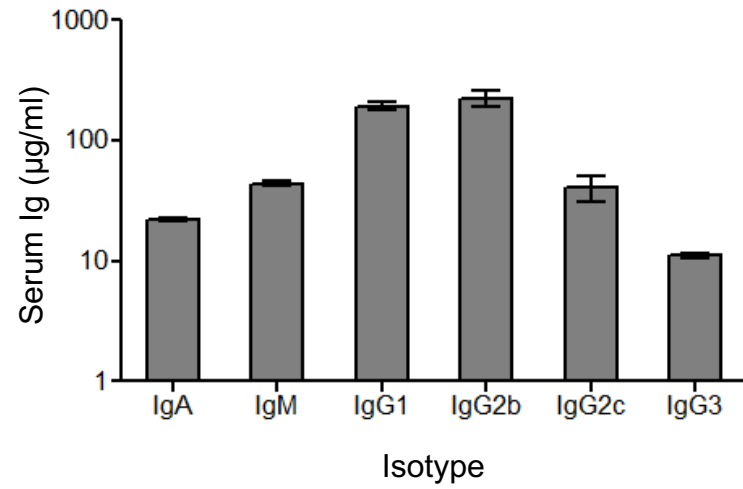

B

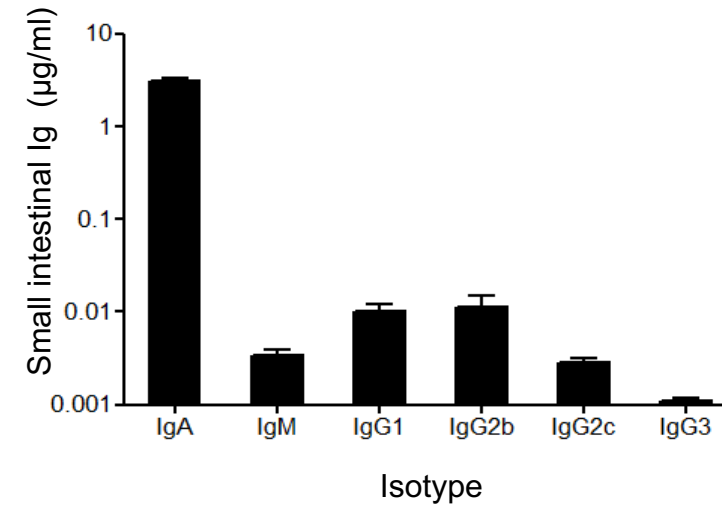

C

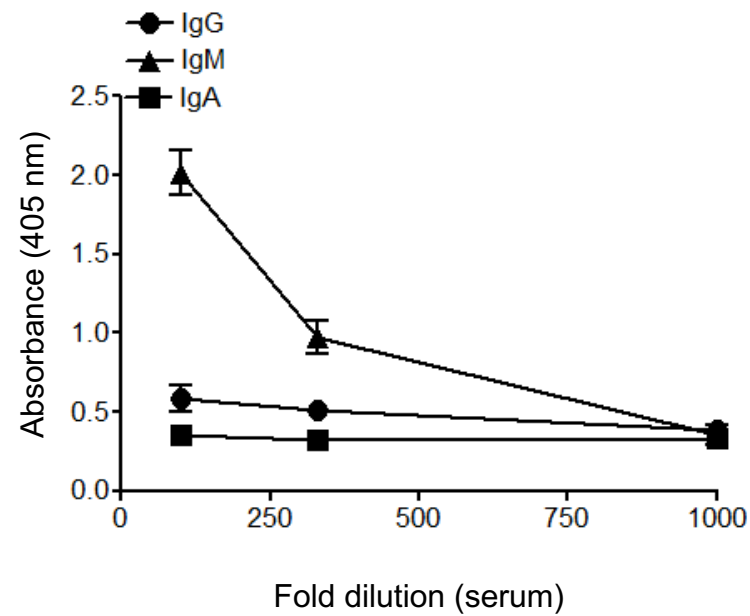

D

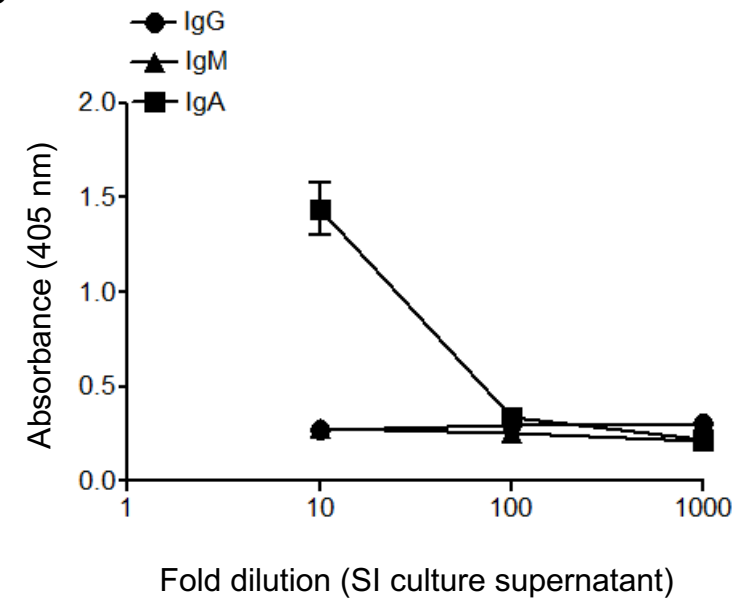

Supplement: FIG S1 [file mBio.02324-19-sf001.pdf]

A

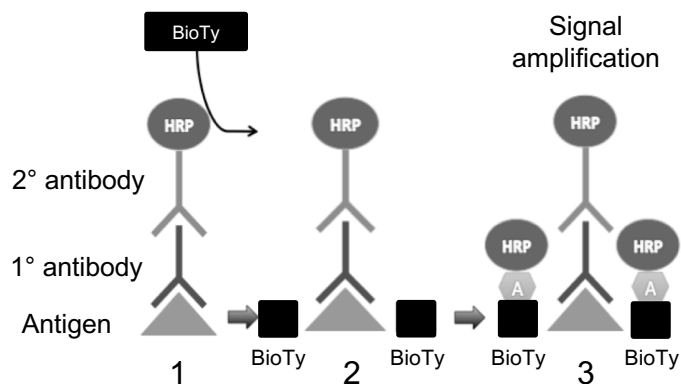

B

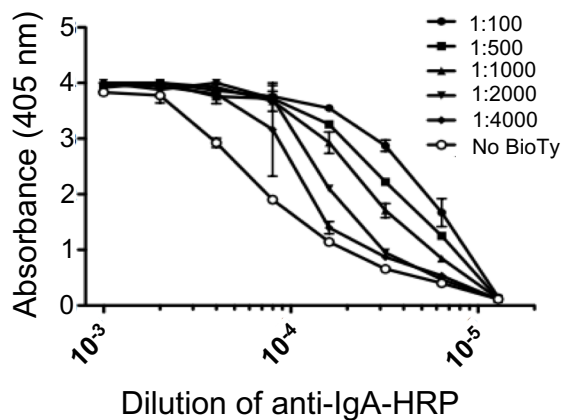

C

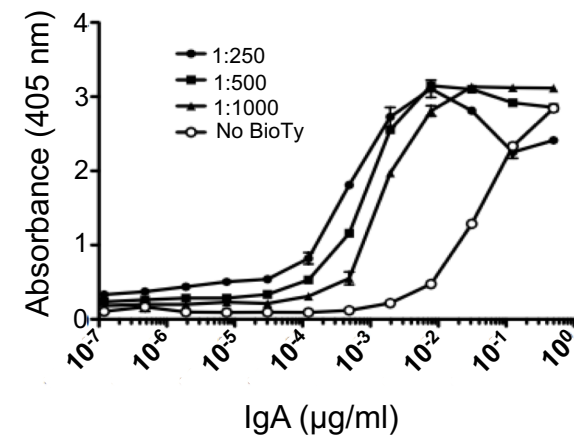

D

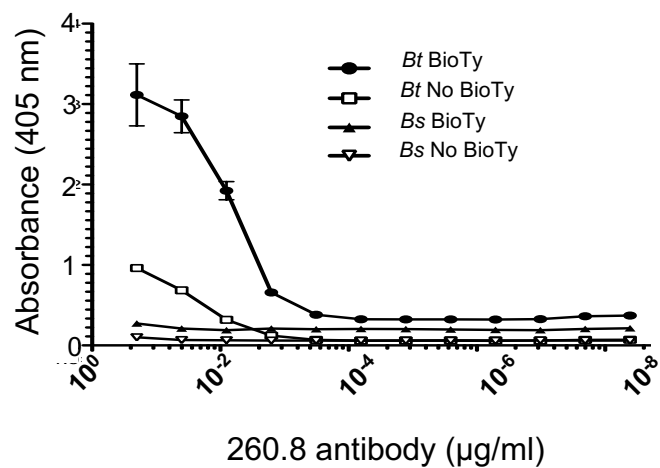

E

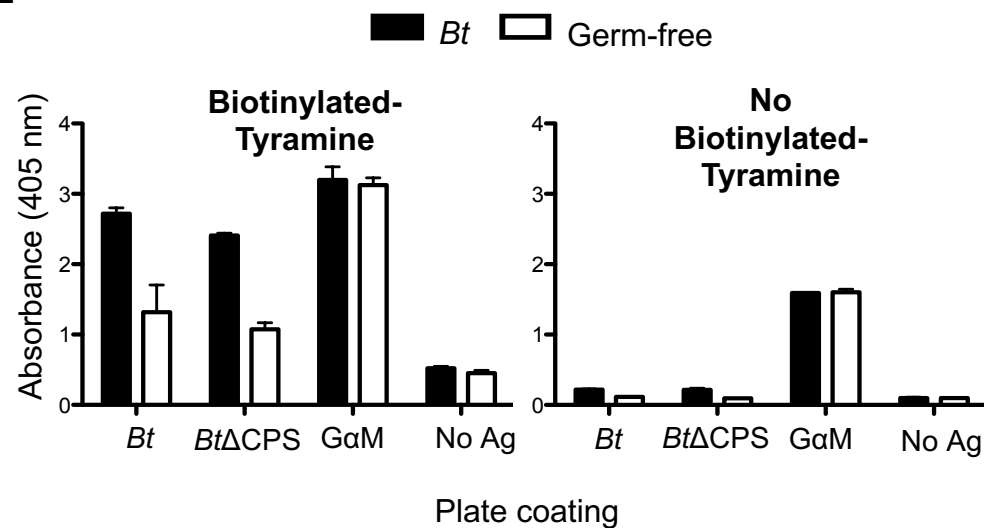

Supplement: FIG S3 [file mBio.02324-19-sf003.pdf]

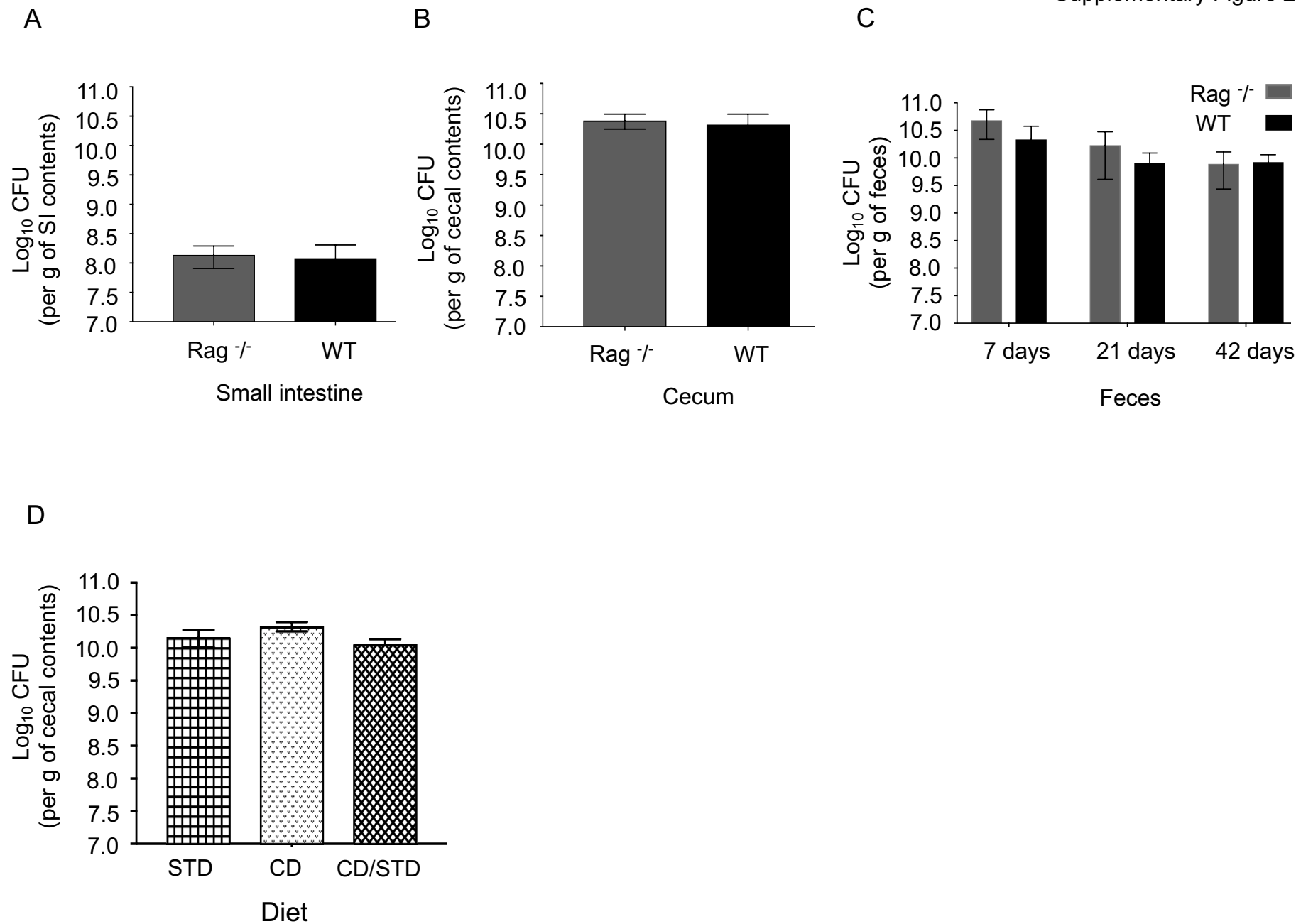

Supplement: FIG S2 [file mBio.02324-19-sf002.pdf]
