# Supplementary material for: Intestinal IgA Regulates Expression of a Fructan Polysaccharide Utilization Locus in Colonizing Gut Commensal Bacteroides thetaiotaomicron
Source: mBio. 2019 Nov 5;10(6):e02324-19. doi: 10.1128/mBio.02324-19 (PMC6831775; doi:10.1128/mBio.02324-19)
Supplement: TEXT S1 [file mBio.02324-19-s0001.pdf]

Supplemental Information (SI)

Materials and methods

Bacterial strains Most studies were carried out using the type strain *Bacteroides thetaiotaomicron* VPI-5482. *Bacteroides thetaiotaomicron* strain 3731, *Bacteroides thetaiotaomicron* strain 7330, *Bacteroides finegoldii* DSM 17565, *Bacteroides ovatus* ATCC 8483*, Bacteroides caccae* ATCC 43185, *Bacteroides fragilis* NCTC 9343*, Bacteroides vulgatus* ATCC 8482, *Parabacteroides merdae* ATCC 43185, *Barnesiella intestinihominis* YIT 11860*, Clostridium cocleatum* ATCC 29901*, Ruminococcus torques* ATCC 27756*, Salmonella enterica* ATCC 14028, *Enterobacter sakazakii*, *Lactobacillus johnsonii*, *Bifidobacterium longum* and *Akkermasia muciniphila* ATCC BAA-835 were used as whole cell antigens for ELISA assays. *Bt* ΔCPS and electrocompetent *E. coli* MegaX DH10B™ (Invitrogen) were used for library construction. A human gut *E.coli* isolate (based on full-length 16S rRNA sequencing) from our lab was used to generate SI culture supernatant that were used as non-specific IgA control.

Sources and Preparation of Carbohydrates Glucose, fructose and levan (# 66674) were all obtained from Sigma.

Colonization of Germ-Free Mice 6-12 weeks old mice germ-free C57BL/6J wild-type (WT) or Rag1$^{-/-}$ mice were housed in gnotobiotic isolators under a strict 12-hour light cycle. Mice were colonized with a single gavage of ~10^8 CFU of wild-type *Bt* grown overnight in TYG (1), and were fed either autoclaved, standard diet (STD) (Labdiet® JL Rat and Mouse/Auto 6F 5K67), or an irradiated mouse diet lacking fructans (Teklad Custom Diet: TD.170584). All experiments were performed using protocols approved by the Johns Hopkins Animal Studies Committee (IACUC).

Generation of Small Intestinal Fragment Lamina Propria Culture Supernatant (SI culture supernatant) Mice were sacrificed using standard aseptic procedures. Small intestine was immediately removed and cleaned using ice-cold DMEM supplemented with 1X Penicillin-Streptomycin and 5% Fetal bovine serum (DMEM-5). Fat tissue was removed, the gut was opened longitudinally and minced into small (2–5 mm) pieces. Intestinal pieces were washed with ice-cold DMEM-5 (3x), followed by ice-cold citrate-buffer solution (50mM tri-sodium citrate, 96mM sodium chloride, 8mM potassium dihydrogen orthophosphate, 5.6mM disodium hydrogen orthophosphate, 1.5mM potassium chloride) (2x). The intestine was then incubated for 15 min on a shaker (~300 rpm) in 30 ml citrate-buffer containing 10% fetal calf serum (CB-FCS) (pre-warmed to 37°C), followed by vortexing for ~ 30 seconds to dislodge intestinal epithelial cells. CB-FCS was removed, and after 3-5 washes with ice-cold DMEM-5, intestinal pieces were placed in a T10 tissue-culture flask with DMEM-10, and incubated for 36 hours at 37°C, 5% CO2. The small intestinal culture supernatant was harvested by centrifugation and filtration (0.2 μM filter) to get rid of cellular debris, and 0.01% sodium azide was added for long term storage at 4°C.

ELISA All ELISAs were performed using standard protocols (2) in 96-well plates. For isotype ELISA, plates were coated overnight at 4°C with goat anti-mouse Ig (Southern Biotech; 1:1500 diluted in sodium bicarbonate coating buffer). Plates were washed with PBS-T (0.05%) (3x) and blocked for 30 min with PBS-BSA (1%) at room temperature. Serial dilutions of serum samples and SI culture supernatants obtained from germ-free or *Bt* mono-colonized mice were added to the 96-well plates, followed by incubation with HRP-conjugated, isotype-specific, secondary antibodies (Southern Biotech; 1:1000

dilution in PBS-BSA (1%)). All steps were carried out either at 4°C overnight, or at room temperature for 2 hours. Plates were developed using ABTS (1 mM; Roche). Reactions were read at a wavelength of 405 nm. Total antibody was quantified using a reference curve obtained from serial dilutions of purified anti-mouse immunoglobulins (Southern Biotech) starting at 1.0 μg/ml.

For antigen specific ELISA, bacterial whole cell antigens were used for coating plates.

For high-sensitivity ELISA, the protocol used was similar to the one described above, expect for modification at the detection step. Briefly, after incubation with HRP-conjugated anti-mouse IgA, 50 μl/well of biotinylated tyramine solution (diluted 1:500 in 0.1 M sodium borate buffer [pH 8.5], 0.1% H_2O_2) was added for signal amplification. Plates were incubated for 15 min at room temperature followed by three washes with PBS-T. Neutralite Avidin-HRP (Southern Biotech; 1:2000 dilution) was added to each well and further incubated for 2 hours at room temperature or overnight at 4°C. Plates were developed as before.

Preparation of Bacterial Whole Cell Antigens Overnight grown bacterial cultures were heat inactivated for 30 min at 60°C, followed by a freeze-thaw cycle and maintained at -80°C.

Preparation of Fecal supernatants Fecal pellets maintained at -80°C were first thawed on ice, and then weighed accurately. A 10% fecal suspension (w/v) was prepared using 1x PBS (pH 7.2), vortexed vigorously for 20 min, followed by centrifugation at 14K rpm for 20 minutes. The resultant supernatant contained IgA that was measured using in high-sensitivity ELISA.

Preparation of Bacterial Whole Cell Lysate *Bt* grown overnight in TYG medium was suspended in 10mM Tris-HCl (pH 8.0) containing 1mM EDTA, 0.1% SDS and bacterial protease inhibitor cocktail (Sigma). Bacterial cells were then lysed by sonication for 3 min using a 10s ON/OFF cycle. Cell debris was removed by centrifugation and protein content of the supernatants was adjusted to 1 mg/ml using Bradford analysis.

Serum Orbital blood was collected from gnotobiotic mice before sacrifice.

Western Blot Analysis Samples were prepared by boiling bacterial whole cell lysate in 2x Laemmli sample buffer (5% β-mercaptoethanol). 0.5 μg of the above lysate was loaded into the wells of 4-20% Mini-PROTEAN TGX™ gel, or 8-16% Criterion TGX™ gel (Bio-Rad) run at a constant voltage of 150V. Proteins were transferred at 100V for 90 min onto a 0.45μm PVDF membrane (GenHunter®). Post blotting, the membrane was incubated at 4°C overnight with polyclonal IgA from diluted SI culture supernatants (100ng/ml) in TBS-BSA. Goat anti-mouse IgA-HRP antibody (Southern Biotech; 1:1500 diluted in TBS-BSA) was used as a secondary antibody. The membrane was developed using Immun-Blot® Opti-4CN™ Colorimetric Kit (Bio-Rad).

For the Proteinase K assay, after protein transfer onto the PVDF membrane, the membrane was incubated with 5 μg/ml Proteinase K for 30 min at 37°C (3).

Construction of *Bt* ΔCPS Genomic Library Random genomic expression library of *Bt* ΔCPS into *E. coli* MegaX DH10B™ T1R Electrocomp™ Cells (Invitrogen) was generated by modifying a previously described protocol (4). Briefly, *Bt* ΔCPS genomic DNA was sheared to a size range of ~2000–5,000 bp by sonication, followed by agarose gel separation and gel purification. Purified DNA was then end-repaired using the End-It™ DNA End Repair kit (Epicentre) and ligated using a Fast-Link™ DNA

Ligation Kit (Epicentre) into pZE21-MCS-1 vector (5), which was linearized at the Hinc II site using inverse PCR (Table S1). 5 μl of dialyzed ligation reaction was used for electroporation. Electroporated cells were inoculated into 10 ml of Luria–Bertani (LB) medium containing 50 μg/ml kanamycin and grown overnight. Overnight cultures were frozen down with 15% glycerol and stored at −80 °C for subsequent screening.

Colony Dot Blot Analysis *Bt* ΔCPS genomic expression library was plated onto LB agar plates containing 50 μg/ml kanamycin and incubated overnight at 37 °C to get ~100 colonies per plate. Colony dot blot assay was performed using a previously published protocol (6).

SI culture supernatant dialysis SI culture supernatants harvested from gnotobiotic mice were added to Amicon Ultra-4 centrifugal filter units with a membrane NMWL of 10 kDa (UFC801024). Filter tubes were centrifuged at 4000 x g for 15 min (Allegra® X-14R series centrifuge, SX4750 swinging bucket rotor) at 25 °C. Sterile PBS (1x) was used for buffer exchange and dialysis of the concentrated SI culture supernatant to get rid of any small molecules, including sodium azide and glucose present in the supernatants. After 3 buffer exchanges, the SI culture supernatant was re-suspended in 1x PBS (same as the starting volume) and stored at 4°C for future use.

***In vitro* growth using SI culture supernatant** Bacteroides minimal medium [100 mM potassium phosphate buffer (pH 7.2), 15 mM NaCl, 4.1 mM cysteine, 7.5mM (NH4)2SO4, 100 μM MgCl2, 1.9 μM hematin, 0.37 nM vitamin B12] supplemented with glucose, fructose or levan (0.5% w/v), and containing SI culture supernatants harvested from gnotobiotic mice (30% v/v) was inoculated (1:50) using a freshly grown culture of

Bt in TYG. Cultures were incubated anaerobically at 37°C and harvested at mid-log phase for RNA isolation.

IgA depletion Dialyzed SI culture supernatants harvested from 6 weeks old C57BL/6J mice were used for IgA depletion. Biotinylated goat anti-mouse IgA (Southern Biotech) was added at a final concentration of 10µg/ml to supernatants containing IgA. Reaction tubes were incubated for 30 min at room temperature. Streptavidin RapidSpheres (EasySep™ Mouse Streptavidin RapidSpheres™ Isolation Kit 19860) were added to the above reaction tube (100 µl/ml) and incubated for 5 min. IgA was depleted using a column free EasySep™ Magnet. Isotype ELISA was performed to confirm selective depletion of IgA.

RNA extraction Bacterial cultures grown *in vitro*, or an aliquot (~ 50-100 mg) of feces/cecal contents was suspended in 2 volumes of RNAprotect (Qiagen) and centrifuged at 3000 x g for 10 min (5424R Eppendorf centrifuge) at room temperature. 500 µl of 2x lysis buffer (200mM NaCl and 20 mM EDTA) was added to the resulting pellet, together with 210 µl of 20% SDS (Ambion) and 500 µl phenol:chloroform:isoamyl alcohol (125:24:1; pH 4.5; Ambion). Acid-washed silica beads (0.1mm zirconia-silica beads 300 mg; BioSpec) were added, and bacteria were lysed using a bead beater (full power for 2 min in the cold room; Mini-Beadbeater by BioSpec). Following centrifugation (13000 x g for 3 min at 4°C), the aqueous layer was subjected to another round of phenol:chloroform:isoamyl alcohol purification, followed by purification with 500 µl of 100% Chloroform. RNA was precipitated by adding 1/10 volume of 3M sodium acetate (pH 5.2; Ambion) plus 1 volume of ice-cold absolute isopropanol. RNA was purified

(RNEasy kit; Qiagen), and residual genomic DNA was removed by DNase treatment (RNase free DNase; Qiagen). Purified RNA was measured using Nanodrop.

cDNA synthesis Quality of RNA extracted from both *in vivo* and *in vitro* sources was confirmed by agarose gel electrophoresis to ensure minimum RNA degradation. cDNA synthesis was carried out using SuperScript® II Reverse Transcriptase (Invitrogen). cDNA reactions were setup containing RNA (10 µl; 100 ng – 1000 ng), random hexamers (1 µl; 250 ngµl^{-1}) and dNTPs (1 µl; 10mM each). Tubes were incubated at 65° C for 5 min, and immediately placed on ice. 1st strand buffer (4 µl; 5x), DTT (2 µl; 0.1 M) and RNase OUT (1 µl; 40 Uml^{-1}) were added to these tubes, which were then placed at 25° C for 2 min. SuperScript® II RT was added (1 µl; 200 Uµl^{-1}) (or RNase free water in no-RT control) and the RT reaction was carried out using T100™ Thermal Cycler by BioRad (25°C for 10min, 42°C for 50 min, 70°C for 15 min). Conversion to cDNA was confirmed by agarose gel electrophoresis.

Quantitative Real Time PCR (qRT-PCR) cDNA was first diluted to 5 ngµl^{-1}assuming a direct stoichiometric conversion of known RNA amount into DNA. This was used in a Brilliant III Ultra-Fast SYBR® Green QPCR reaction (Qiagen) to measure gene transcript levels (Primes - Table S1). Diluted cDNA (2 µl) was added to a qRT-PCR reaction containing 2× SYBR Green (10 µl), forward primer (0.5 µl; 20µM), reverse primer (0.5 µl; 20µM) and Nuclease free water (7 µl). Assays were performed with specific primers in technical replicates using Bio-Rad CFX96 instrument. At least 3 biological replicates were used for each reaction. Reaction cycle used for the reaction was: 3 min at 95°C (1x); 5 sec at 95°C followed by 5 sec at 60°C (40x); melt curve. Relative gene expression was calculated using the ΔΔCT method with E correction.

Development of High-sensitivity ELISA assay

The study of commensal-specific intestinal IgA has been challenging given that traditional immunoassays are not very sensitive in detecting it directly in fecal samples. This may be explained by presence of interfering agents in samples that could inhibit the assays, and by low concentration of specific IgA in these samples. For our current study we observed that even in a highly simplified monocolonized setup, presence of polyclonal IgA in fecal and small intestinal culture supernatants made quantification of specific IgA against *Bt*'s protein antigens very challenging. To circumvent this issue, we developed a low cost and high-sensitivity ELISA assay that significantly improved the detection of antigen-specific IgA present in our samples.

The assay is a modification of a traditional horseradish peroxidase (HRP) based ELISA (7). The initial steps are identical to a classical ELISA, where the surface of a 96-wells ELISA plate is coated with an antigen of interest that allows capture of antigen-specific antibodies (here IgA) present in feces or small intestinal culture supernatants.

Classically, presence of IgA is then detected by oxidation of a colorimetric substrate using an anti-IgA conjugated-HRP antibody. We modified this last step using a previously published *catalyzed reporter deposition* (CARD) method (**Supplementary Figure 3A**) (8). The method relies on the presence of low concentrations of HRP (as used in signal amplification reactions), which catalyze oxidative condensation of phenolic compounds such as biotinylated-tyramine that then bind electron-rich moieties of proteins. This results in the deposition of large amounts of biotinylated-tyramine near antigen-antibody complexes. Multiple molecules of biotinylated-tyramine can be bound in this manner, which allows for signal amplification. Every molecule of biotinylated-

tyramine can then capture a streptavidin-linked HRP. These HRP molecules are ultimately used to catalyze the oxidation of the colorimetric substrate 2,2'-azino-bis(3-ethylbenzthiazoline-6-sulfonic acid) (ABTS). The deposition of additional HRP thus enables signal amplification and improves the detection limit of the assay.

We carried out a number of tests to standardize the conditions of the assay and confirm that amplification occurs without compromising specificity. As shown in **Supplementary Figure 3B**, for a given concentration of purified IgA, presence of biotinylated-tyramine amplified the anti-IgA-HRP signal by >2 absorbance units within the linear range. This amplification resulted in an improved sensitivity and allowed detection of purified IgA present at a concentration that was 100 times lower than that could be detected without tyramine amplification (**Supplementary Figure 3C**). To confirm that the amplification is not at the cost of specificity, we carried out our assay using serial dilutions of culture supernatants from hybridoma cells secreting *Bt* specific monoclonal IgA (260.8) that served as a primary antibody (**Supplementary Figure 3D**) (6). Data shows amplification of signal only in wells coated with *Bt* whole cell lysate, and not when a non-specific antigen such as *Bacteroides sartorii* that was used for antibody capture.

We then tested our assay using feces and small intestinal supernatants (data for feces shown here) that were generated from germ-free and *Bt* monocolonized mice. As shown in **Supplementary Figure 3E**, tyramine amplification increased the signal significantly, and allowed detection of *Bt*-specific IgA response in feces derived from *Bt* monocolonized mice. Similar to Figure 1 in the main text, we again observed a low-level reactivity of germ-free fecal IgA against *Bt* antigens. We also used purified, individual *Bt*

capsular polysaccharides (cps) to detect capsular antigen-specific IgA in a polyclonal IgA mixture present in *Bt* monocolonized mice feces (unpublished data).

These results demonstrate the utility of our assay in measuring gut bacteria specific IgA, when it is present at levels that are below the detection limit offered by traditional ELISA.

Supplementary references

1. **Sonnenburg JL, Xu J, Leip DD, Chen CH, Westover BP, Weatherford J, Buhler JD, Gordon JI.** 2005. Glycan foraging in vivo by an intestine-adapted bacterial symbiont. Science **307:**1955-1959.
2. **Peterson DA, McNulty NP, Guruge JL, Gordon JI.** 2007. IgA response to symbiotic bacteria as a mediator of gut homeostasis. Cell Host Microbe **2:**328-339.
3. **Macpherson AJ, Gatto D, Sainsbury E, Harriman GR, Hengartner H, Zinkernagel RM.** 2000. A primitive T cell-independent mechanism of intestinal mucosal IgA responses to commensal bacteria. Science **288:**2222-2226.
4. **Forsberg KJ, Patel S, Gibson MK, Lauber CL, Knight R, Fierer N, Dantas G.** 2014. Bacterial phylogeny structures soil resistomes across habitats. Nature **509:**612-616.
5. **Lutz R, Bujard H.** 1997. Independent and tight regulation of transcriptional units in Escherichia coli via the LacR/O, the TetR/O and AraC/I1-I2 regulatory elements. Nucleic Acids Res **25:**1203-1210.

6. **Peterson DA, Planer JD, Guruge JL, Xue L, Downey-Virgin W, Goodman AL, Seedorf H, Gordon JI.** 2015. Characterizing the interactions between a naturally primed immunoglobulin A and its conserved Bacteroides thetaiotaomicron species-specific epitope in gnotobiotic mice. J Biol Chem **290:**12630-12649.
7. **Engvall E, Perlmann P.** 1971. Enzyme-linked immunosorbent assay (ELISA). Quantitative assay of immunoglobulin G. Immunochemistry **8:**871-874.
8. **Bobrow MN, Litt GJ, Shaughnessy KJ, Mayer PC, Conlon J.** 1992. The use of catalyzed reporter deposition as a means of signal amplification in a variety of formats. J Immunol Methods **150:**145-149.
