# Supplementary material for: Intestinal IgA Regulates Expression of a Fructan Polysaccharide Utilization Locus in Colonizing Gut Commensal Bacteroides thetaiotaomicron
Source: mBio. 2019 Nov 5;10(6):e02324-19. doi: 10.1128/mBio.02324-19 (PMC6831775; doi:10.1128/mBio.02324-19)
Supplement: TABLE S1 [file mBio.02324-19-st001.pdf]

Supplemental Information (SI)

Table S1. List of primers used in this study.

**Primer**	**Sequence**
**pZE-21 MCS-1 linearization**	
pZE linear F	GACGGTATCGATAAGCTTGAT
pZE linear R	GACCTCGAGGGGGGG
**pZE-21MCS-1**<br>**multiple cloning site**<br>**(for sequencing)**	
pZE MCS F	CCCCCCCTCGAGGTC
pZE MCS R	ATCAAGCTTATCGATACCGTC
**qPCR primers**	
BT1757 F	GTCTTTCAAACGCTGCAACA
BT1757 R	GAGCTTCGGGAACAGACTTG
BT1763 F	ATGCCTGGTCACCTACGAAC
BT1763 R	CAAGCGGTCCATTCTCATTT
BT16S F	GTGTAGCGGTGAAATGCTTAGATATC
BT16S R	CAGTGTCAGTTGCAGTCCAGTGA
