# Supplementary material for: Intestinal IgA Regulates Expression of a Fructan Polysaccharide Utilization Locus in Colonizing Gut Commensal Bacteroides thetaiotaomicron
Source: mBio. 2019 Nov 5;10(6):e02324-19. doi: 10.1128/mBio.02324-19 (PMC6831775; doi:10.1128/mBio.02324-19)
Supplement: TABLE S2 [file mBio.02324-19-st002.pdf]

Supplemental Information (SI)

Table S2. Genes encoded by the *Bt* ΔCPS genomic library inserts carrying putative epitopes recognized by polyreactive gut IgA

**Locus tag(s) within a genomic insert**	**Predicted molecular mass (kDa)**	**Predicted function**	**Predicted protein localization**
BT0189; BT0190	38; 127	anti-sigma factor; SusC homolog	P; M
BT1161; BT1162; BT1163	53; 46; 27	aminopeptidase C; hypothetical; TolC family	Ex; M; M
BT1212	51	efflux protein	M
BT1268; BT1269; BT1270	112; 40; 47	AcrB/D/F family transporter; component of multidrug efflux system; putative Na+/H+ antiporter (part of polyamine transport locus (1))	M; Cy; M
BT1286; BT1287; BT1288	65; 24; 52	two-component system response regulator; hypothetical; spermidine/putrescine ABC	M, Ex; M

		transporter (part of polyamine transport locus (1))	
BT1385; BT1386; BT1387	34; 21; 49	transcriptional regulator; hypothetical; putative Na+-driven multidrug efflux pump	Cy; Cy; M
BT1438; BT1439; BT1440	52; 55.46; 115	membrane efflux protein; SusD homolog; SusC homolog	M; M; M
BT1760; BT1761; BT1762	59; 50; 64	Glycoside hydrolase 32; SusE homolog; SusD homolog (2)	M; M; M
BT2437	23	hypothetical	M
BT3013; BT3014; BT3015	73; 33;107	SusD homolog; chitobiase; hypothetical	M; M; Unknown
BT3152; BT3153; BT3154; BT3155;	57; 13; 12; 90	ATP-dependent DNA helicase; hypothetical; hypothetical; Glycosyl hydrolase 65	Cy; Cy; M; Ex
BT3328; BT3329; BT3330	95; 69; 38	hypothetical; hypothetical; hypothetical	M; M; M
BT3411; BT3412; BT3413	76; 22, 53	pyrophosphate-energized vacuolar membrane proton	M; Cy; M

		pump; RNase HII; hypothetical	
BT3669; BT3670	21; 117	Hypothetical; SusC homolog	P; M
BT3967; BT3968; BT3969	41; 34;161	putative two-component system sensor; Cation efflux; AcrB/D/F family cation efflux	M; Ex; M
BT4152; BT4153	113; 48	beta-galactosidase I; Exopolygalacturonase precursor	Ex; Ex
BT4191; BT4192; BT4193	48; 31; 84	Hypothetical; lipoyl synthase; dipeptidyl-peptidase IV	M; Cy; Ex
BT4675; BT4676; BT4677; BT4678	42; 16; 19; 44	heparin lyase I precursor; putative periplasmic protein; hypothetical; L-serine ammonia-lyase	Ex; Ex; Ex; Cy

M: membrane; Ex: Extracellular; Cy: Cytoplasmic; P: Periplasmic

Supplementary references

1. **Xu J, Bjursell MK, Himrod J, Deng S, Carmichael LK, Chiang HC, Hooper LV, Gordon JI.** 2003. A genomic view of the human-Bacteroides thetaiotaomicron symbiosis. Science **299:**2074-2076.

2. **Sonnenburg ED, Zheng H, Joglekar P, Higginbottom SK, Firbank SJ, Bolam DN, Sonnenburg JL.** 2010. Specificity of polysaccharide use in intestinal bacteroides species determines diet-induced microbiota alterations. Cell **141:**1241-1252.
