# Supplementary material for: Intestinal IgA Regulates Expression of a Fructan Polysaccharide Utilization Locus in Colonizing Gut Commensal Bacteroides thetaiotaomicron
Source: mBio. 2019 Nov 5;10(6):e02324-19. doi: 10.1128/mBio.02324-19 (PMC6831775; doi:10.1128/mBio.02324-19)
Supplement: TABLE S3 [file mBio.02324-19-st003.pdf]

Supplemental Information (SI)

Table S3. Composition of Teklad Custom Diet (TD.170584) used in the study.

**Formula**	**g/Kg**
Casein	207.00
DL-Methionine	3.00
Dextrose, anhydrous	325.12
Corn Starch	320.00
Corn Oil	50.00
Cellulose	50.00
Mineral Mix, Ca-P Deficient (79055)	13.37
Calcium Phosphate, dibasic	17.50
Calcium Carbonate	4.00
Vitamin Mix, Teklad (40060)	10.00
Ethoxyquin, antioxidant	0.01

Standard Diet: Labdiet® JL Rat and Mouse/Auto 6F 5K67 (https://www.labdiet.com/)
